# The Influence of Ankle Mobility and Foot Stability on Jumping Ability and Landing Mechanics: A Cross-Sectional Study

**DOI:** 10.3390/jfmk9030160

**Published:** 2024-09-08

**Authors:** Antonino Patti, Marco Gervasi, Valerio Giustino, Flavia Figlioli, Alberto Canzone, Patrik Drid, Ewan Thomas, Giuseppe Messina, Domenico Savio Salvatore Vicari, Antonio Palma, Antonino Bianco

**Affiliations:** 1 Sport and Exercise Sciences Research Unit, Department of Psychology, Educational Science and Human Movement, University of Palermo, Via Giovanni Pascoli 6, 90144 Palermo, Italy; antonino.patti01@unipa.it (A.P.); flavia.figlioli@unipa.it (F.F.); alberto.canzone98@gmail.com (A.C.); ewan.thomas@unipa.it (E.T.); domenicosaviosalvatore.vicari@univr.it (D.S.S.V.); antonio.palma@unipa.it (A.P.); antonino.bianco@unipa.it (A.B.); 2 Department of Biomolecular Sciences, Division of Exercise and Health Sciences, University of Urbino Carlo Bo, 61029 Urbino, Italy; marco.gervasi@uniurb.it; 3 Faculty of Sport and Physical Education, University of Novi Sad, 21000 Novi Sad, Serbia; patrikdrid@gmail.com; 4 Department of Human Sciences and Promotion of the Quality of Life, San Raffaele University, 20132 Rome, Italy; giuseppe.messina@uniroma5.it; 5 Department of Neurosciences, Biomedicine and Movement Sciences, University of Verona, 37129 Verona, Italy; 6 Regional Sports School of CONI Sicilia, 90141 Palermo, Italy

**Keywords:** ankle injury, postural control, range of motion, balance

## Abstract

Practicing physical activities or sports that involve frequent jumping and landing can strain the muscles and joints of the lower limbs, especially in those who do not have adequate physical preparation. The objective of this study was to (a) determine the correlation between ankle range of motion (ROM) and landing stability following jumps; (b) assess the association between the jump height in a counter-movement jump (CMJ) test and ankle ROM; and (c) examine the connection between stabilometry during specific jumps movements present in many sports and in ankle stabilization. Sixty-two healthy amateur volleyball players participated in the study (age: thirty-seven females and twenty-five males; age (years): 16.5 ± 4.25; height (cm): 166 ± 11.4; weight (Kg): 61.6 ± 13.7). Participants were recruited for the study in collaboration with student sports associations. The evaluations encompassed the measurement of ankle joint mobility for both lower limbs using an inertial sensor, a static baropodometric and stabilometric analysis using a pressure platform, and the CMJ test using the Microgate system. After the assessments, participants performed a “specific jump landing task”. Significant correlations were found between foot range of motion (ROM) and counter-movement jump (CMJ) performance. Specifically, the ROM of the right foot had a strong correlation with the CMJ (r = 0.81, *p* < 0.001), while the left foot ROM showed a moderate correlation (r = 0.46, *p* < 0.001). The specific jump task revealed substantial changes in stabilometry parameters, particularly during forward hops compared to lateral jumps. Dorsiflexion ROM significantly impacts jumping ability. Evaluating landing patterns and stabilometry during targeted activities can help optimize training, improve dynamic balance, and reduce ankle injury risk.

## 1. Introduction

In many sports and sports activities, repeated jumps and landings place significant stress on the lower limb muscles and joints, increasing the risk of musculoskeletal injuries [1]. In a study, Bahr et al. reported that in 78% of the cases, team players had a history of at least one prior ankle injury [2,3]. In a population of volleyball players, the authors showed that ankle injuries primarily happened during the act of landing after blocking, whereas most other injuries were linked to take-off, landing, or the spiking motion during an attack [3]. However, noteworthy relationships are also present in other sports gestures. Akbari H, et al. (2023) investigated the relationship between the ROM of ankle landing positions during a soccer-specific task [4]. The results showed that a reduced ROM of ankle dorsiflexion was associated with greater landing errors in a soccer-specific situation. For this reason, it could be possible to state that the assessment of ankle mobility is an important process to prevent faulty movements and potentially related injuries [4]. Similar athletic gestures are observed in other sports as well. Ungureanu AN, Beratto L. et al. demonstrated that rugby players also exhibit comparable kinematics. Their study revealed performance improvements, particularly in jumping, sprinting, and high-intensity running [5].

These risks are heightened in amateur sports and student leagues, where participants are at a greater risk of sustaining non-contact injuries [6]. Asperti AM et al. demonstrated that injury rates are significantly higher in amateur sports, particularly those focused on fitness improvement or student activities [6]. This can lead to prolonged interruptions or even withdrawal from physical activity. Adolescent sports injuries present a significant issue that can lead to withdrawal from physical activity. Further research is needed to better understand the impact of risk factors and improve prevention efforts [7].

The literature suggests the need for preventive strategies and training programs, but further research into the etiology of injuries is essential to develop effective measures. Injuries to the ankle and knee joints, particularly those to the anterior cruciate ligament (ACL), have been linked to ankle joint kinematics, such as dorsiflexion angles during landings [8,9,10].

In 2021, Cejudo A. presented evidence indicating potential disparities in range of motion (ROM) between genders. This study provides gender-specific scores for lower extremity flexibility profiles in basketball players. This study suggests that athletic trainers and conditioning coaches identify players who may be at high risk of injury due to abnormal ROM scores [11]. The study of Boden et al. (2009) evaluated the foot position and hip, knee, and ankle joint angles of athletes at the time of an ACL injury and compared these values with a control group of athletes who experienced no injuries [8]. The results of this study showed that athletes of the control group first contacted the ground with the forefoot while athletes of the experimental group had first ground contact with the hindfoot or entirely flatfooted.

Another study by Malloy et al. (2016) analyzed the association between ankle dorsiflexion flexibility and landing kinematics in female soccer players during a drop vertical jump [12]. The results showed that females with less ankle dorsiflexion flexibility exhibited greater peak knee abduction moments and angles as well as less peak knee flexion angles during landing, suggesting that ankle dorsiflexion may influence landing posture kinematics and kinetics, making its evaluation important for injury prevention [12]. A limitation of this study declared by the authors is that the ankle dorsiflexion was evaluated with the knee in the full extended position, and in this way, it was not possible to measure the soleus muscle, which can influence the dorsiflexion flexibility [12]. Another limitation of this study is the height of the box that was normalized and not weighted to the height of the subject. We hypothesize that the landing height concerning the anthropometric measurements of the tested subject are variable and should not be overlooked and indefinitely personalizing the evaluation task as much as possible is necessary. Other studies have also noted limitations such as not simulating sport-specific tasks or standardizing drop jump box heights [13,14]. The lateral cutting movements are very frequent in team sports like volleyball [15]. Over time, these pressures might result in persistent ankle instability caused by harm to the lateral ankle ligaments [16]. However, there are limited studies on stability strategies during unilateral jump-landing tasks [17].

Another interesting study showed that increasing the ROM of the ankle, particularly dorsiflexion, in addition to preventing injuries, showed an increase in performance on the single-leg vertical jump height of fifty-two recreational athletes of both genders [18]. Rabin, A. et al. also confirmed this relationship in their study, highlighting the importance of evaluating ankle dorsiflexion [19].

The objective of this study was to examine the connection between ankle range of motion (ROM) and landing stability following jumps, evaluate the correlation between ankle ROM and jump height in the counter-movement jump (CMJ) test, and analyze stabilometry during specific jumping movements and landing by introducing a customizable task tailored to the subject’s characteristics.

## 2. Materials and Methods

### 2.1. Study Design

This is a cross-sectional study. Given the study’s purpose, a cross-sectional design was deemed more appropriate, as it is useful for assessing the prevalence of conditions, behaviors, or outcomes and is well suited for evaluating associations between variables by analyzing multiple outcomes.

### 2.2. Subjects

**Figure 1:**
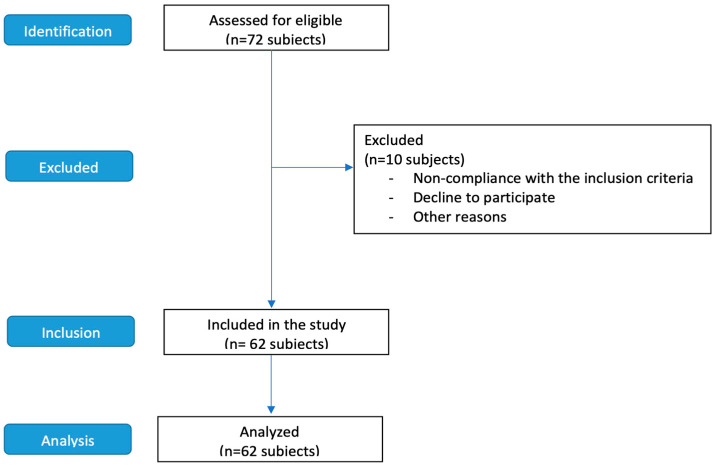
Experimental design: STROBE Flow diagram.

This study recruited a total of seventy-two students who play volleyball at an amateur level. However, only sixty-two individuals (thirty-seven females and twenty-five males; age (years): 16.5 ± 4.25; height (cm): 166 ± 11.4; weight (Kg): 61.6 ± 13.7; dominant foot (n): right 55 subjects and left 7 subjects) fulfilled the inclusion requirements. The remaining 10 were excluded due to not having played or exercised regularly for at least six months (Table 1). An a priori sample size power analysis with an α error of 0.05 and an effect size of 0.5 revealed that sixty-two participants would be sufficient to reach a power of 99% [20]. The STROBE guidelines were used to ensure a high-quality presentation of the conducted observational study (Figure 1) [21]. Consistent with the recommendations of the literature and comparable research [10,22,23,24], we included in the study those who met the following inclusion criteria:

(a) Participants had no leg injuries in the past six months.
(b) All participants had been playing and exercising regularly for at least six months.
(c) There were no post-surgical subjects.

Researchers collected demographics and sports injury history, and participants signed written informed consent forms. Parental consent was also obtained for minors (<18 years). The subjects were invited to the gymnasium, where they were briefed on the research and evaluations without disclosing the study’s objectives to avoid influencing performance. Anthropometric data were collected from the same research unit between September 2022 and December 2022. We also obtained written informed consent from parents of minors (<18 years). The study was carried out in compliance with the principles of the Declaration of Helsinki and approved by the Bioethics Committee of the University of Palermo (n. 94/2022-Prot. 70310; 4 July 2022).

### 2.3. Procedures

Prior to the evaluations, participants engaged in a standardized warm-up regimen consisting of gentle jogging and a combination of static and dynamic stretching exercises to prime the muscles and joints. The evaluations were carried out in the team gymnasium on a conventional wooden floor from 5:00 to 8:00 PM. All participants were required to have a minimum recovery interval of 72 h since their last game to ensure they were not fatigued. The initial evaluation focused on assessing ankle joint mobility by measuring both the range and quality of movement in each ankle. Participants then performed a counter-movement jump (CMJ). Following 30 min of rest, participants had stabilometric analysis, which was especially conducted during the jump-landing phase to evaluate their balance and postural control in dynamic situations. This analysis offered valuable perspectives on the post-landing stability management strategies.

#### 2.3.1. The Measure of the Ankle Range of Motion

**Figure 2:**
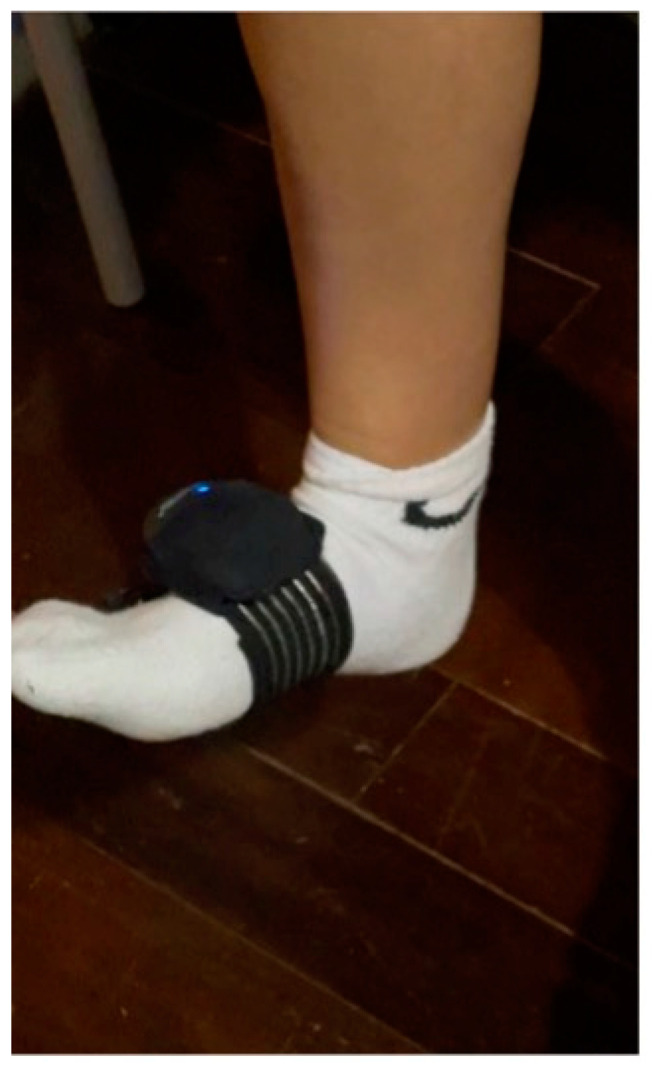
Measurement of joint mobility with accelerometer.

One small Bluetooth sensor was used to measure the joint mobility of the ankle (internal sampling up to 1000 Hz; Bluetooth 4.0 and 2.0—USB connection 2.0; weight: 28 gr; dimensions: 65 × 45 × 18 mm; resolution: accelerometer = ±2 G to ±16 G gyroscope = ±200°/s to ±2000°/s magnetometer = ±4000 μT). BEYOND Inertial used the Beyond framework software (Motustech SRL, Guidonia Montecelio, Roma, Italy). BEYOND Inertial was attached to the foot’s dorsum of each participant using ad hoc straps [25] to limit their oscillations relative to the underlying segment [26] (Figure 2). Once the accelerometer was set, the subject, who was in a sitting position with flexed knee, was asked to actively perform a maximum plantar flexion movement and a maximum back flexion movement. In line with similar studies, we recorded ankle ROM after a single evaluation, following familiarization sessions in previous days. This approach aimed to replicate the natural ankle adaptation during game phases, where multiple flexions typically do not occur before a jump [27].

Data acquired by the BEYOND Inertial were transmitted via Bluetooth to a laptop. The parameters considered for the statistical analysis were as follows:

- Range of motion (ROM°): It represents the angular excursion carried out by the segment that rotates from its starting point to its arrival point.
- Angular speed (°/s): It represents the average angular velocity over the entire range of motion.
- Fluency Index: An index ranging from 0 to 1 indicates the movement’s quality. The closer it is to 1, the smoother the movement. The dorsiflexion, plantar flexion, eversion, and inversion of both feet of all the subjects analyzed were evaluated on these three parameters (Figure 2).

#### 2.3.2. Counter-Movement Jump (CMJ)

The Microgate system (Bolzano, Italy) was used to manage this test. The system allows for the quantity of flight and contact times during the execution of a series of jumps, with a precision of 1/1000 of a second. It is an optical detection system constituted of a transmitting and a receiving bar starting from these fundamental basic data; the dedicated software allows for obtaining a series of parameters related to performance with maximum precision and in real time [28]. After fully explaining experimental procedures, subjects completed a warm-up consisting of running (5 min), stretching of lower extremity muscles, and submaximal vertical jumping for the familiarization (3 min) [29]. For the test, athletes began from the upright standing position with their hands on the hips; they flexed their knees (about 90°) as fast as possible and then leaped as high as possible in the next maximum extensory phase [29]. Subjects were to leave the floor with knees and ankles extended and land in an extended position. Three measurements were administered per subject, 2 min were allowed between jump repetitions, and the best performance was considered for the study analysis [30].

#### 2.3.3. Specific Jump-Landing Task

The study published by Butler, R. J. et al. inspired the method for performing this test [31]. However, it was adapted to suit the specific requirements of subjects engaged in sporting activities. In this modified test, athletes jumped from the ground to a stabilometric platform positioned at a distance equal to half their height. An obstacle set at 70% of their previously recorded CMJ performance was placed between the take-off point and the platform. This adaptable modification was designed to individualize testing based on each athlete’s performance level, more closely replicating sporting situations. The subjects landed on the platform with only one leg seeking the best stabilization in the shortest possible time. Participants performed single-leg landings on the platform and were instructed to stabilize for 20 s post-landing, during which stabilometric data were recorded [32]. The tests included one-legged landings after forward jumps for both legs and lateral jumps (side-step cutting) for both legs. Participants were required to adhere to predefined conditions regarding platform distance and obstacle height for all jumps. A 120 s rest period was provided between trials, and each trial was repeated three times, with the best performance on single-leg landings selected for analysis. Throughout the tests, participants’ hands remained free. A trial was considered a failure if the athlete did not maintain balance for the required 20 s, jumped or moved the affected foot on the platform, dismounted with the opposite limb, or landed with the affected foot not fully on the platform [32]. The 20 s duration was chosen based on the study by Fransz, D. P. et al., which identified a stabilization period of 3–5 s following a single-leg jump. However, we adopted the maximum duration analyzed by the authors [33]. These tests were administered using the FreeMed system (FreeStep v.1.0.3 software, Sensor Medica, Guidonia Montecelio, Roma, Italy). The following parameters of the statokinesigram were considered: sway path length of the center of pressure (CoP) (mm); ellipse surface area (mm^2^); coordinates of the CoP along the frontal (X; right–left; x-mean) and sagittal (Y; forward–backward; y-mean) planes; and maximum oscillation and average speed of movement (mm/s) [34]. The platform’s sensors are 24 K gold, allowing for high reliability.

### 2.4. Statistical Analyses

All data were recorded in an Excel file. Statistical analysis was performed with Jamovi (2.3.21.0).

The distribution of quantitative data was assessed with the Shapiro–Wilk test (*p* > 0.05). For the objectives of the study, the Pearson correlation test (r) was used to analyze the relationship among the dorsiflexion, the plantarflexion, and the eversion and inversion of the right and left feet with the entire battery of tests used. The r values are distributed as follows: r = 0.10–0.29, small or low correlation; r = 0.30–0.49, medium or moderate correlation; r = 0.50–1.0, large or high or strong correlation [35]. The independent samples *t*-test was used to compare and evaluate performance differences between female and male subjects. Paired samples *t*-test was used to evaluate if there are differences between the performance of the left leg and the right leg and between the landing after a forward jump right vs. left on jump-landing task. A multiple linear regression was used with the vertical jump height as dependent variables and the right and left dorsiflexion ROM, the right and left plantarflexion ROM foot size, right and left sway path length, and average speed of movement of both feet as independent variables that could be predictors. Statistical significance was set a priori at *p* < 0.05.

## 3. Results

Table 1 presents the demographic description. Table 2, Table 3, Table 4 and Table 5 describe the correlation between the ankle valuation (right and left) and other parameters. A correlation was present between the right foot ROM° vs. vertical jump height (r = 0.81, *p* < 0.001) and the left foot ROM° vs. vertical jump height (r = 0.46, *p* < 0.001; Table 2). The analysis of dates showed interesting results, particularly with the foot’s dorsiflexion. Table 6 is a comparison of parameters between males and females using the independent samples *t*-test. Data analysis showed differences in jumping performance.

Furthermore, the multiple linear regression showed a significant regression with the dependent variable vertical jump height (cm) and the independent variables’ Right Dorsiflexion Range of Mov. °, foot size, right sway path length, right average speed of movement, and Right Plantarflexion Range of Mov.° (regression *p*-value < 0.001; adjusted R^2^ = 0.078; Table 7). A similar tendency was found on the left foot with a significant regression with the dependent variable vertical jump height (Cm) and the independent variable: foot size, left sway path length, left average speed of movement, and Left Plantarflexion Range of Mov. ° (regression *p*-value < 0.001; adjusted R^2^ = 0.33 (Table 8)). In addition, we analyzed the differences between the subjects who claimed to be with the right dominant foot (55 subjects) vs. left dominant foot (7 subjects), but no significant differences in dominant foot interaction were present. The specific task volley test showed significant differences between the left ellipse surface area after a forward jump vs. the left ellipse surface area after a lateral jump (884.86 ± 304.7 mm^2^ vs. 1056.91 ± 386.1 mm^2^; *p* < 0.001); between the left average speed of movement after a forward jump vs. the left average speed of movement after a lateral jump (44.82 ± 16 mm/s vs. 54.25 ± 20.9 mm/s; *p* < 0.001); and between the left y-mean after a forward jump vs. left y-mean after a lateral jump (12.13 ± 16.6 mm vs. 7.19 ± 16; *p* < 0.05). The test did not show significant differences on the right foot (Table 9).

## 4. Discussion

This study aimed to identify predictive indicators of future instability using a specific task-test jump. Correlations between ankle range of motion (ROM) and the specific jump-landing task (SJLT) yielded interesting findings. Dorsiflexion parameters were particularly significant in predicting chronic ankle instability [36]. In line with the results of Donovan et al., a reduced range of motion correlates with decreased strength, impaired neuromuscular control, and altered functional movement patterns [36].

Our results showed that the Dorsiflexion Fluency Index of the right foot, which is an index ranging from 0 to 1 and which indicates the movement quality of the ankle, significant inverse correlation with stability parameters of the foot after a one-legged landing on a forward jump. Stability is represented by the size of the ellipse surface area [37]. In addition, this parameter showed a significative positive correlation with the x-mean parameter after a one-legged landing after a forward jump and on a lateral jump both on the right foot and left foot. The x-mean parameter represented the coordinates of the CoP along the frontal planes [38]. These findings are along the same lines as the conclusions showed by Brown et al. [39]. The authors demonstrated that the mechanically unstable subjects displayed differences in frontal plane ankle motion [39]. In 2023, Han et al. provided insights into ankle dorsiflexion ranges, distinguishing between Hypo-DFROM (below 40 degrees), Normal-DFROM, and Hyper-DFROM [40]. Our results described a mean of ROM° below 40 degrees in the whole sample analyzed. None of the subjects enrolled in the study reported chronic ankle problems; this indicates the importance of regular and ongoing assessments of the ankle in athletes to prevent latent ankle impairments and future injuries. Furthermore, the data showed by Han et al. confirm our conclusions on how limited dorsiflexion negatively affects the landing/cutting task [40].

Regarding limb dominance, our study found no significant differences, though left-foot dominant subjects were underrepresented and warrant further investigation. However, significant differences were observed in performance after the specific jump-landing task, particularly in the left limb. After lateral jumps, the left foot showed a significantly greater sway path length, average speed of movement, and lower y-mean, indicating greater difficulty in stabilization compared to the right foot. Our results seem to indicate that the left foot is more difficult to stabilize on a one-legged landing task after a lateral jump than the right foot. These results are in line with the conclusions of the study by Simpson, J.D. et al. (2018) [17]. The authors demonstrated that individuals with chronic ankle instability showed dynamic postural stability deficits and reduced neuromuscular control during unilateral jump-landings [17].

Our results also highlighted reduced ankle sagittal plane displacement after lateral jumps compared to forward jumps (Table 9), a strategy observed in subjects with chronic ankle instability to reduce impact forces on the ankle complex [39,41]. As suggested by the literature, the reduction in ankle sagittal plane range is an ankle strategy during the post-landing period that decreases impact forces imposed on the ankle complex, but a greater reliance is transmitted to the proximal segments [42,43]. Furthermore, our data analysis demonstrated a significant correlation between dorsiflexion ROM and performance on the counter-movement jump (CMJ) test, particularly with the right leg (Table 2). In 2021, Panoutsakopoulos V. et al. found similar results, the authors hypothesized that individuals with a larger ankle dorsiflexion angle can more efficiently utilize the additional work provided by the arm swing in the vertical squat jump compared to individuals with a less flexible ankle joint [44].

Multiple linear regression analysis further confirmed dorsiflexion as a predictor of vertical jump height, with significant associations observed for variables such as right sway path length and average speed of movement (Table 7). Similar trends were observed for the left leg but to a lesser extent (Table 8).

The study is not without limitations. Some subjects were slightly older than eighteen, which may have introduced greater heterogeneity to the sample; the BEYOND Inertial has demonstrated reliability, and similar instruments of lower technical specifications have undergone extensive validation [25]. However, while its previous version is well-documented in the literature [45,46], the validation of the current version is still in progress. Moreover, the sample analyzed specifically consisted of students who played volleyball on an amatorial level. Nevertheless, we were unable to evaluate the impact of prior years of playing experience on the individuals. Insufficient control over the prior experience of each participant may have resulted in a certain level of variability in the findings. Thus, it is recommended that future studies give priority to selecting a more homogeneous sample with similar levels of experience in physical exercise to reduce the impact of this factor. To validate the reliability of the findings, it would be beneficial to expand the parameters for selecting participants or include a mandatory minimum number of years of expertise as a controlled factor.

## 5. Conclusions

This study is the first to propose a jump-landing task that tries to simulate real sports movement to be customized for specific anthropometric characteristics and performance. This may show altered adaptation strategies during a landing/cutting activity like in the play actions. Dorsiflexion ROM has a very high influence on jumping performance, and exercises to improve ankle joint mobility are essential not only to prevent injuries but also for the performance itself. Future studies should investigate the connections between lower limb movement patterns, neuromuscular control, and joint kinematics to gain a better understanding of the causes of recurring lateral ankle sprains in populations that frequently engage in repetitive jump-landings. These studies are particularly important in amateur and student settings, where injury rates are higher, and could help reduce post-injury dropout from physical activity.

## Figures and Tables

**Table 1:** Subject demographics.

	Subjects (n)	Age (y)	Height (cm)	Weight (kg)
Mean	F (37)	16.0	161	55.9
	M (25)	17.2	174	69.9
Standard deviation	F (37)	4.78	8.57	11.7
	M (25)	3.27	10.6	12.4

M: Male; F: female; n: numbers; y: years; cm: centimeters; Kg: kilograms.

**Table 2:** Correlation between the dorsiflexion of the foot and all parameters.

			Dorsiflexion of Foot					
			Left Foot			Right Foot		
			Angular Speed (°/s)	Range of Motion (ROM°)	Fluency Index	Angular Speed (°/s)	Range of Motion (ROM°)	Fluency Index
Left foot	Dorsiflexion	Angular speed (°/s)						
		Range of motion (ROM°)						
		Fluency Index	0.400 **					
	Plantarflexion	Angular speed (°/s)	0.360 **		0.275 *		−0.281 *	
		Range of motion (ROM°)						
		Fluency Index	0.540 ***		0.487 ***	0.356 **		
	Eversion	Angular speed (°/s)	0.378 **		0.261 *	0.443 ***		0.293 *
		Range of motion (ROM°)						0.250 *
		Fluency Index			0.395 **	0.334 **		
	Inversion	Angular speed (°/s)	0.449 ***			0.597 ***		0.287 *
		Range of motion (ROM°)						
		Fluency Index	0.422 ***					
Right foot	Dorsiflexion	Angular speed (°/s)			0.334 **			
		Range of motion (ROM°)		0.538 ***				
		Fluency Index			0.355 **	0.403 **		
	Plantarflexion	Angular speed (°/s)	0.308 *			0.585 ***		0.345 **
		Range of motion (ROM°)		0.452 ***				
		Fluency Index	0.321 *		0.267 *	0.312 *	−0.287 *	0.262 *
	Eversion	Angular speed (°/s)	0.294 *		0.280 *	0.627 ***		0.494 ***
		Range of motion (ROM°)		0.274 *			0.322 *	0.355 **
		Fluency Index			0.339 **	0.309*		0.251 *
	Inversion	Angular speed (°/s)				0.647 ***		0.337 **
		Range of motion (ROM°)				0.381 **		
		Fluency Index			0.308 *	0.267 *		0.263 *
Left foot	One-legged landing after a forward jump	Ellipse Surface Area (mm^2^)				−0.315 *		−0.406 **
		Maximum oscillation						
		Average speed of movement (mm/s)						
		X-mean (mm)			0.363 **	0.308 *		0.530 ***
		Y-mean (mm)						
Right foot	One-legged landing after a forward jump	Ellipse Surface Area (mm^2^)						−0.310 *
		Maximum oscillation				0.254 *		
		Average speed of movement (mm/s)						
		X-mean (mm)						0.305 *
		Y-mean (mm)						
Left foot	One-legged landing on a lateral jump	Ellipse Surface Area (mm^2^)						−0.352 **
		Maximum oscillation						
		Average speed of movement (mm/s)						
		X-mean (mm)			0.279 *			
		Y-mean (mm)						
Right foot	One-legged landing on a lateral jump	Ellipse Surface Area (mm^2^)						
		Maximum oscillation						
		Average speed of movement (mm/s)						
		X-mean (mm)						
		Y-mean (mm)						
		CMJ		0.464 ***			0.810 ***	

The threshold for significant differences between performances is defined as: * *p* < 0.05, ** *p* < 0.01, *** *p* < 0.001.

**Table 3:** Correlation between the plantarflexion of the foot and all parameters.

			Plantarflexion of Foot					
			Left Foot			Right Foot		
			Angular Speed (°/s)	Range of Motion (ROM°)	Fluency Index	Angular Speed (°/s)	Range of Motion (ROM°)	Fluency Index
Left foot	Dorsiflexion	Angular speed (°/s)						
		Range of motion (ROM°)						
		Fluency Index						
	Plantarflexion	Angular speed (°/s)						
		Range of motion (ROM°)						
		Fluency Index	0.483 ***					
	Eversion	Angular speed (°/s)	0.428 ***		0.382 **			
		Range of motion (ROM°)		0.399 **				
		Fluency Index	0.355 **		0.403 **			
	Inversion	Angular speed (°/s)	0.405 **		0.453 ***			
		Range of motion (ROM°)		0.499 ***				
		Fluency Index			0.303 *			
Right foot	Dorsiflexion	Angular speed (°/s)						
		Range of motion (ROM°)						
		Fluency Index						
	Plantarflexion	Angular speed (°/s)	0.466 ***		0.436 ***			
		Range of motion (ROM°)		0.585 ***				
		Fluency Index	0.478 ***		0.464 **	0.626 ***		
	Eversion	Angular speed (°/s)	0.379 **	0.280 *	0.320 *	0.715 ***		0.380 **
		Range of motion (ROM°)		0.303 *			0.290 *	
		Fluency Index			0.290*	0.378 **		0.359 **
	Inversion	Angular speed (°/s)	0.252 *		0.300*	0.656 **		0.497 ***
		Range of motion (ROM°)		0.377 **		0.270 *	0.309 *	
		Fluency Index			0.369 **	0.298 *		
Left foot	One-legged landing after a forward jump	Ellipse Surface Area (mm^2^)						
		Maximum oscillation						
		Average speed of movement (mm/s)						
		X-mean (mm)						
		Y-mean (mm)						
Right foot	One-legged landing after a forward jump	Ellipse Surface Area (mm^2^)						
		Maximum oscillation						
		Average speed of movement (mm/s)						
		X-mean (mm)						
		Y-mean (mm)						
Left foot	One-legged landing on a lateral jump	Ellipse Surface Area (mm^2^)						
		Maximum oscillation						
		Average speed of movement (mm/s)						
		X-mean (mm)						
		Y-mean (mm)						
Right foot	One-legged landing on a lateral jump	Ellipse Surface Area (mm^2^)						
		Maximum oscillation						
		Average speed of movement (mm/s)						
		X-mean (mm)						
		Y-mean (mm)						
		CMJ						

The threshold for significant differences between performances is defined as: * *p* < 0.05, ** *p* < 0.01, *** *p* < 0.001.

**Table 4:** Correlation between the eversion of the foot and all parameters.

			Eversion of Foot					
			Left Foot			Right Foot		
			Angular Speed (°/s)	Range of Motion (ROM°)	Fluency Index	Angular Speed (°/s)	Range of Motion (ROM°)	Fluency Index
Left foot	Dorsiflexion	Angular speed (°/s)						
		Range of motion (ROM°)						
		Fluency Index						
	Plantarflexion	Angular speed (°/s)						
		Range of motion (ROM°)						
		Fluency Index						
	Eversion	Angular speed (°/s)						
		Range of motion (ROM°)						
		Fluency Index	0.579 ***					
	Inversion	Angular speed (°/s)	0.684 ***		0.352 **			
		Range of motion (ROM°)		0.307 *				
		Fluency Index	0.298 *		0.255 *			
Right foot	Dorsiflexion	Angular speed (°/s)						
		Range of motion (ROM°)						
		Fluency Index						
	Plantarflexion	Angular speed (°/s)	0.671 ***		0.585 ***			
		Range of motion (ROM°)		0.314 *				
		Fluency Index	0.489 ***		0.465 ***			
	Eversion	Angular speed (°/s)	0.695 ***		0.380 **			
		Range of motion (ROM°)		0.629 ***				
		Fluency Index	0.314 *		0.480 ***	0.464 ***		
	Inversion	Angular speed (°/s)	0.673 ***		0.461 ***	0.637 ***		0.300 *
		Range of motion (ROM°)				0.347 **		
		Fluency Index	0.462 ***		0.433 ***	0.288 *		0.459 ***
Left foot	One-legged landing after a forward jump	Ellipse Surface Area (mm^2^)						
		Maximum oscillation						
		Average speed of movement (mm/s)						
		X-mean (mm)						
		Y-mean (mm)						
Right foot	One-legged landing after a forward jump	Ellipse Surface Area (mm^2^)						
		Maximum oscillation						
		Average speed of movement (mm/s)						
		X-mean (mm)						
		Y-mean (mm)						
Left foot	One-legged landing on a lateral jump	Ellipse Surface Area (mm^2^)						
		Maximum oscillation						
		Average speed of movement (mm/s)						
		X-mean (mm)						
		Y-mean (mm)						
Right foot	One-legged landing on a lateral jump	Ellipse Surface Area (mm^2^)						
		Maximum oscillation						
		Average speed of movement (mm/s)						
		X-mean (mm)						
		Y-mean (mm)						
		CMJ						

The threshold for significant differences between performances is defined as: * *p* < 0.05, ** *p* < 0.01, *** *p* < 0.001.

**Table 5:** Correlation between the inversion of the foot and all parameters.

			Inversion of Foot					
			Left Foot			Right Foot		
			Angular Speed (°/s)	Range of Motion (ROM°)	Fluency Index	Angular Speed (°/s)	Range of Motion (ROM°)	Fluency Index
Left foot	Dorsiflexion	Angular speed (°/s)						
		Range of motion (ROM°)						
		Fluency Index						
	Plantarflexion	Angular speed (°/s)						
		Range of motion (ROM°)						
		Fluency Index						
	Eversion	Angular speed (°/s)						
		Range of motion (ROM°)						
		Fluency Index						
	Inversion	Angular speed (°/s)						
		Range of motion (ROM°)						
		Fluency Index	0.382 **					
Right foot	Dorsiflexion	Angular speed (°/s)						
		Range of motion (ROM°)						
		Fluency Index						
	Plantarflexion	Angular speed (°/s)	0.633 ***		0.265 *			
		Range of motion (ROM°)		0.476 ***				
		Fluency Index	0.413 ***					
	Eversion	Angular speed (°/s)	0.684 ***	0.290 *				
		Range of motion (ROM°)						
		Fluency Index						
	Inversion	Angular speed (°/s)	0.757 ***					
		Range of motion (ROM°)	0.298 *	0.657 ***		0.288 *		
		Fluency Index				0.36 **		
Left foot	One-legged landing after a forward jump	Ellipse Surface Area (mm^2^)						
		Maximum oscillation						
		Average speed of movement (mm/s)						
		X-mean (mm)						
		Y-mean (mm)						
Right foot	One-legged landing after a forward jump	Ellipse Surface Area (mm^2^)						
		Maximum oscillation						
		Average speed of movement (mm/s)						
		X-mean (mm)						
		Y-mean (mm)						
Left foot	One-legged landing on a lateral jump	Ellipse Surface Area (mm^2^)						
		Maximum oscillation						
		Average speed of movement (mm/s)						
		X-mean (mm)						
		Y-mean (mm)						
Right foot	One-legged landing on a lateral jump	Ellipse Surface Area (mm^2^)						
		Maximum oscillation						
		Average speed of movement (mm/s)						
		X-mean (mm)						
		Y-mean (mm)						
		CMJ						

The threshold for significant differences between performances is defined as: * *p* < 0.05, ** *p* < 0.01, *** *p* < 0.001.

**Table 6:** Comparison of parameters between males and females using the independent samples *t*-test.

Description			Gender	n	Mean	SD	*p*	Cohen’s d
Age			F	37	16.027	4.775	ns	
			M	25	17.240	3.270		
Height			F	37	160.730	8.568	<0.001	−1.47
			M	25	174.080	10.571		
Weight			F	37	55.946	11.723	<0.001	−1.16
			M	25	69.880	12.364		
Left foot	One-legged landing after a forward jump	* Ellipse Surface Area (mm^2^)	F	37	67,044.45	36,620.1	ns	
			M	25	82,007.7	66,993.9		
		* Sway path length (mm)	F	37	845.427	281.710	ns	
			M	25	943.225	333.231		
		* Maximum oscillation	F	37	42.056	63.484	0.007	−0.61
			M	25	99.247	125.736		
		* Average speed of movement (mm/s)	F	37	42.774	14.492	ns	
			M	25	47.860	17.984		
		* X-mean	F	37	−31.809	13.256	ns	
			M	25	−30.845	18.738		
		Y-mean	F	37	9.493	14.314	ns	
			M	25	16.026	19.085		
Right foot	One-legged landing after a forward jump	* Ellipse Surface Area (mm^2^)	F	37	77,004.3	38,035.4	ns	
			M	25	85,871.6	48,083.5		
		* Sway path length (mm)	F	37	923.166	349.061	ns	
			M	25	876.222	284.824		
		* Maximum oscillation	F	37	95.330	152.405	ns	
			M	25	72.798	132.586		
		* Average speed of movement (mm/s)	F	37	46.846	17.650	ns	
			M	25	45.978	18.437		
		X-mean	F	37	−29.909	11.828	ns	
			M	25	−28.670	12.373		
		Y-mean	F	37	10.631	10.952	ns	
			M	25	12.533	16.041		
Left foot	One-legged landing on a lateral jump	* Ellipse Surface Area (mm^2^)	F	37	72,284.882	38,690.435	ns	
			M	25	74,317.066	32,100.34		
		* Sway path length (mm)	F	37	1090.288	442.459	ns	
			M	25	1007.506	284.450		
		* Maximum oscillation	F	37	76.547	110.790	ns	
			M	25	75.708	120.193		
		* Average speed of movement (mm/s)	F	37	55.658	23.406	ns	
			M	25	52.162	16.778		
		* X-mean	F	37	−32.728	25.125	ns	
			M	25	−35.727	15.995		
		Y-mean	F	37	5.659	17.210	ns	
			M	25	9.467	14.178		
Right foot	One-legged landing on a lateral jump	* Ellipse Surface Area (mm^2^)	F	37	69,621.044	37,242.685	ns	
			M	25	79,571.328	77,463.133		
		Sway path length (mm)	F	37	1034.607	331.264	0.027	0.58
			M	25	868.578	186.447		
		* Maximum swing	F	37	101.488	144.359	ns	
			M	25	51.333	60.789		
		* Average speed of movement (mm/s)	F	37	52.636	175.341	ns	
			M	25	43.969	9.822		
		* X-mean	F	37	−27.628	17.750	ns	
			M	25	−29.159	27.710		
		* Y-mean	F	37	7.660	9.008	ns	
			M	25	10.980	21.543		
Left foot	Dorsiflexion	* Angular speed (°/s)	F	37	44.838	34.791	ns	
			M	25	42.800	23.272		
		Range of motion (ROM°)	F	37	29.754	5.913	ns	
			M	25	30.896	6.269		
		* Fluency Index	F	37	0.86	0.156	ns	
			M	25	0.88	0.116		
Left foot	Plantarflexion	* Angular speed (°/s)	F	37	46.703	36.325	ns	
			M	25	47.520	45.710		
		Range of motion (ROM°)	F	37	44.935	9.012	ns	
			M	25	46.088	11.211		
		* Fluency Index	F	37	0.83	0.142	ns	
			M	25	0.82	0.145		
Left foot	Eversion	Angular speed (°/s)	F	37	47.730	25.941	ns	
			M	25	40.040	21.255		
		Range of motion (ROM°)	F	37	32.205	9.936	ns	
			M	25	32.824	7.925		
		* Fluency Index	F	37	0.866	0.141	ns	
			M	25	0.858	0.134		
Left foot	Inversion	* Angular speed (°/s)	F	37	54.757	22.869	ns	
			M	25	47.920	19.455		
		Range of motion (ROM°)	F	37	37.970	8.495	ns	
			M	25	39.092	11.572		
		* Fluency Index	F	37	0.907	0.098	ns	
			M	24	0.838	0.206		
Right foot	Dorsiflexion	* Angular speed (°/s)	F	37	45.973	27.046	ns	
			M	25	45.640	20.512		
		Range of motion (ROM°)	F	37	29.827	6.193	ns	
			M	25	31.520	6.754		
		* Fluency Index	F	37	0.860	0.120	ns	
			M	25	0.917	0.107		
Right foot	Plantarflexion	* Angular speed (°/s)	F	37	50.351	25.588	ns	
			M	25	41.840	20.134		
		Range of motion (ROM°)	F	37	43.543	8.861	ns	
			M	25	42.776	9.620		
		* Fluency Index	F	37	0.843	0.116	ns	
			M	25	0.824	0.101		
Right foot	Eversion	* Angular speed (°/s)	F	37	51.054	27.993	ns	
			M	25	48.640	28.110		
		Range of motion (ROM°)	F	37	33.030	8.463	ns	
			M	25	34.380	12.373		
		* Fluency Index	F	37	0.879	0.183	ns	
			M	25	0.863	0.125		
Right foot	Inversion	* Angular speed (°/s)	F	37	53	25.970	ns	
			M	25	48	22.856		
		Range of motion (ROM°)	F	37	37.924	8.3194	ns	
			M	25	42.056	13.204		
		* Fluency Index	F	37	0.869	0.176	ns	
			M	25	0.901	0.105		
CMJ			F	37	18.735	4.805	0.001	−0.89
			M	25	23.324	5.609		

*: U of Mann-Whitney analysis; ns: not significant, SD: Standard deviation.

**Table 7:** Multiple linear regression (dependent variable: Performance CMJ (Cm); independent variable: Right Dorsiflexion Range of Mov.°, foot size, right sway path length, right average speed of movement, and Right Plantarflexion Range of Mov.°). Regression *p*-value < 0.001; Adjusted R^2^ = 0.78.

Multiple Linear Regression—CMJ (cm)				
Predictor	Estimate	SE	t	*p*
Intercept	−24.694	6.143	−4.019	<0.001
Right Dorsiflexion Range of Mov. °	0.674	0.057	11.672	<0.001
Foot size	0.605	0.127	4.767	<0.001
Right sway path length (mm)	0.017	0.006	2.890	0.005
Right average speed of movement (mm/s)	−0.322	0.107	−3.018	0.004
Right Plantarflexion Range of Mov.°	−0.006	0.041	−0.145	0.885

**Table 8:** Multiple linear regression (dependent variable: Performance CMJ (Cm); independent variable: Left Dorsiflexion Range of Mov.°, foot size, left sway path length, left average speed of movement and Left Plantarflexion Range of Mov.°). Regression *p*-value < 0.001; Adjusted R^2^ = 0.33.

Multiple Linear Regression—CMJ (cm)				
Predictor	Estimate	SE	t	*p*
Intercept	−16.919	9.644	−1.754	0.085
Left Dorsiflexion Range of Mov. °	0.489	0.108	4.545	<0.001
Foot size	0.634	0.211	3.007	0.004
Left sway path length (mm)	0.002	0.017	0.102	0.919
Left average speed of movement (mm/s)	−0.066	0.327	−0.200	0.842
Left Plantarflexion Range of Mov.°	−0.031	0.065	−0.471	0.639

**Table 9:** Pair *t*-test between landing after a forward jump right vs. left and on a lateral jump (l).

Measurements			*p*
Left ellipse surface area (mm^2^)	vs.	Right ellipse surface area (mm^2^)	0.147
Left sway path length (mm)	vs.	Right sway path length (mm)	0.603
Left maximum oscillation	vs.	Right maximum swing	0.363
Left x-mean	vs.	Right x-mean	0.305
Left y-mean	vs.	Right y-mean	0.655
Left ellipse surface area (mm^2^)	vs.	(l)—Left ellipse surface area (mm^2^)	0.997
Left sway path length (mm)	vs.	(l)—Left sway path length (mm)	<0.001
Left maximum oscillation	vs.	(l)—Left maximum oscillation	0.589
Left average speed of movement (mm/s)	vs.	(l)—Left average speed of movement (mm/s)	<0.001
Left x-mean	vs.	(l)—Left x-mean	0.420
Left y-mean	vs.	(l)—Left y-mean	0.014
Right ellipse surface area (mm^2^)	vs.	(l)—Right ellipse surface area (mm^2^)	0.313
Right sway path length (mm)	vs.	(l)—Right sway path length (mm)	0.154
Right maximum swing	vs.	(l)—Right maximum oscillation	0.839
Right average speed of movement (mm/s)	vs.	(l)—Right average speed of movement (mm/s)	0.288
Right x-mean	vs.	(l)—Right x-mean	0.671
Right y-mean	vs.	(l)—Right y-mean	0.146

## Data Availability

The data presented in this study are available on request from the corresponding author.
